# The Daily Press and Medical Societies

**Published:** 1886-07

**Authors:** 


### THE DAILY PRESS AND MEDICAL SOCIETIES.

At a recent meeting of the Chicago Medical Society, a motion was made to rescind the standing resolution, under which reporters for the daily papers are excluded. The motion was rejected for substantially the same reasons that led the Society to pass the original resolution. It was then claimed that reporters sought chiefly sensational matters, and that subjects of scientific interest were ignored, and the Society and its proceedings were placed in an unfavorable light before the public.

The resolution has proved inoperative, for in many instances, when anything sensational has occurred, the gist of the matter has appeared in the morning papers in a more or less garbled and unsatisfactory manner. The reporters gather their information about these matters as best they can, either from interviews with members of the Society or otherwise.

Since much of the proceedings of the Society is of interest to the public, as well as to the medical profession, if properly reported, it would seem to be well to admit reliable reporters to the meetings of the Society, and it would seem to be practicable to have such persons detailed for that duty.

